# A Mobile Health Intervention to Reduce Pain and Improve Health (MORPH) in Older Adults With Obesity: Protocol for the MORPH Trial

**DOI:** 10.2196/resprot.9712

**Published:** 2018-05-14

**Authors:** Jason Fanning, Amber K Brooks, Edward Ip, Barbara J Nicklas, W Jack Rejeski

**Affiliations:** ^1^ Department of Health and Exercise Science Wake Forest University Winston-Salem, NC United States; ^2^ Section on Geriatric Medicine Department of Internal Medicine Wake Forest School of Medicine Winston-Salem, NC United States; ^3^ Department of Anesthesiology Wake Forest School of Medicine Winston-Salem, NC United States; ^4^ Department of Biostatistical Sciences Wake Forest School of Medicine Winston-Salem, NC United States

**Keywords:** technology, weight loss, sedentary lifestyle, chronic pain, mindfulness

## Abstract

**Background:**

Chronic pain is a complex, age-related health issue that affects both physical functioning and quality of life. Because the impact of chronic pain is worsened by obesity and inactivity, nonpharmacological interventions that promote movement, reduce sitting, and aid in weight loss are needed to help manage pain symptoms among older adults with chronic pain.

**Objective:**

The Mobile Intervention to Reduce Pain and Improve Health (MORPH) pilot trial aims to develop and test the feasibility and acceptability of a novel, patient-centered intervention to reduce chronic pain and improve physical functioning in older adults, leveraging the combination of telecoaching and individually adaptive mHealth tools to decrease both body mass and sedentary behavior.

**Methods:**

MORPH comprises 2 phases, including a 1-year iterative development phase, and a 1-year pilot randomized controlled trial (RCT). During the development phase, representative participants will engage in one-on-one structured interviews and a 1-week field test. The resulting feedback will be used to guide the development of the finalized MORPH intervention package. During the second phase, the finalized intervention will be tested in a pilot RCT (N=30) in which older adult participants with chronic pain and obesity will be assigned to receive the 12-week MORPH intervention or to a waitlist control. Primary outcomes include self-reported pain symptoms and physical function.

**Results:**

Phase 1 recruitment is ongoing as of December 2017.

**Conclusions:**

The MORPH intervention brings together a strong body of evidence using group-based behavioral intervention designs with contemporary mHealth principles, allowing for intervention when and where it matters the most. Given the ubiquity of smartphone devices and the popularity of consumer activity and weight monitors, the results of this study may serve to inform the development of scalable, socially driven behavioral pain management interventions.

**Trial Registration:**

ClinicalTrials.gov NCT03377634; https://clinicaltrials.gov/ct2/show/NCT03377634 (Archived by WebCite at http://www.webcitation.org/6yj0J5Pan)

**Registered Report Identifier:**

RR1-10.2196/9712

## Introduction

Chronic pain has emerged as an urgent age-related [1] health issue, significantly impacting one’s physical functioning [2] and quality of life [3], often causing individuals to withdraw from meaningful social relationships [4]. Pain is a complex health concern with symptoms that vary across the day. Whereas the long-term prescription of opioids is a common means of managing these symptoms, it has resulted in an epidemic of opioid misuse: more than 37% of drug-overdose deaths in 2013 were due to pharmaceutical opioids [5]. Finally, it is a costly disability: the annual cost of pain in the United States is nearly 30% higher than the costs of cancer and diabetes combined [6].

Similar to pain, obesity impacts the older adult population broadly such that 1 in 3 older adults is obese [7]. Unfortunately, pain becomes more common with increasing body mass index (BMI; [8]), and obesity exacerbates many of the adverse health effects of chronic pain. Obesity contributes to increased pain severity [9], with abdominal obesity nearly doubling the risk for chronic pain in older adults [10], and it negatively impacts the musculoskeletal system [11] by increasing mechanical stress on the body [12], inducing physical limitations and bodily pain. In fact, higher daily ratings of pain severity are associated with decreased physical performance in older adults [13].

**Figure 1:**
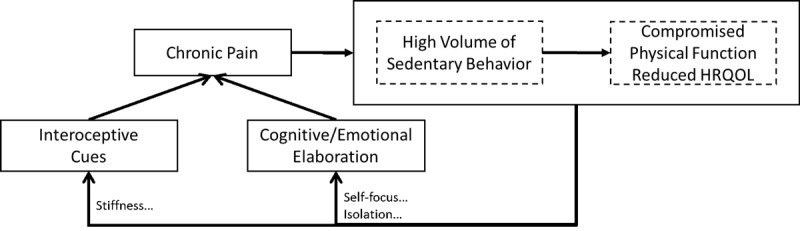
Pathways between chronic pain, sedentary behavior, and compromised physical function and health-related quality of life (HRQOL).

The experience of pain varies across the day [14]; it is complex and multidimensional [15]. This experience is marked by an interaction between interoceptive cues and the cognitive and emotional responses that modulate the perception of pain. Pain perceptions can contribute to decrements in physical function [13] and withdrawal from physical [16] and social activities [4]; these then feed back onto both interoceptive cues, cognition, and emotion (see Figure 1). Previous evidence suggests that targeting cognitive and emotional responses can buffer the pain response [17-19], and brief mindfulness-based breathing exercises have been shown to ameliorate acute pain responses [20]. Given that chronic pain interferes with behavioral self-management [21], we expect that cueing these brief exercises (see [20]) alongside light to moderate movement at key moments (ie, before peaks in pain and affective lows; see the section titled “Individually Adaptive Content”) will be instrumental in supporting lasting behavior change in this population.

We suggest that promoting light to moderate physical activity across the day will be important for improving pain and reducing weight in this population for several reasons. Long bouts of sedentary behavior (SB) negatively impact metabolic health independent of participation in moderate to vigorous physical activity (MVPA [22,23]), and intervening on SB by promoting postural shifts and the accumulation of activity across the day is effective for promoting long-term weight loss in older adults [24]. This approach to reducing SB results in a larger total volume of physical activity relative to MVPA promotion while producing fewer aversive physical sensations and is often preferable to older adults and those with compromised function [23,25]. Still, intervening on SB carries unique challenges. Individuals must be made aware of their patterns of activity across the day, and in turn must regularly apply strategies to address unhealthy patterns of sitting (ie, the presence of long, unbroken SB bouts).

**Figure 2:**
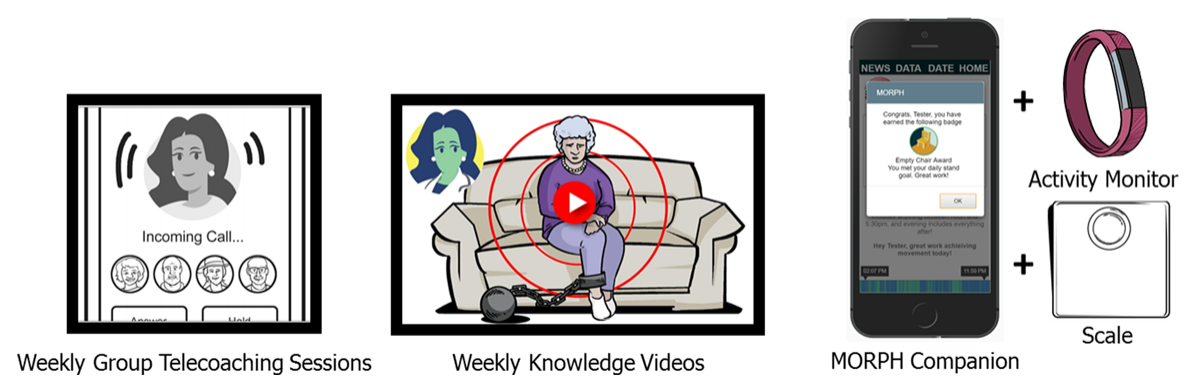
Key Mobile Intervention to Reduce Pain and Improve Health (MORPH) intervention components.

Given the unique challenges faced by older adults with chronic pain, intervention strategies are needed that can provide real-time intervention content to support changes in diet and activity patterns across the day. These methods need to tailor content to individuals’ daily pain experiences, bring the intervention to the participant, and help to combat social isolation. Fortunately, advances in consumer technologies—including the widespread use of smartphone and tablet devices across demographic groups [26] and increased popularity of consumer monitoring devices—for the first time facilitate this type of immersive, home-based intervention targeting sedentary and diet behaviors across the day. The purpose of the Mobile Intervention to Reduce Pain and Improve Health (MORPH) is to develop and test the feasibility of an innovative and broadly scalable theory- and evidence-driven group telecoaching and mHealth intervention that aims to reduce SB while promoting day-long movement and dietary weight loss. The MORPH intervention comprises 3 core components: (1) weekly group telecoaching sessions; (2) highly engaging weekly animated educational videos; and (3) MORPH Companion, a package of mHealth tools that together target weight loss and SB with the aim of reducing pain and enhancing physical function in overweight/obese adults with chronic pain (see Figure 2).

## Methods

A description of the 2-phase MORPH study follows. Study protocols were reviewed and approved by a university institutional review board in February, 2018, and all participants will complete a written informed consent.

### Phase I: Development of MORPH

MORPH is a 12-week weight loss and SB telecoaching intervention that is supported by the MORPH Companion mHealth package, and the content of MORPH is described in the following sections. The MORPH study will be subdivided into 2 phases. The first phase is dedicated to the development of MORPH Companion, as it is the primary vehicle of delivery for the intervention. Broadly, the design of the MORPH Companion app is grounded in social cognitive theory [27] and the Ritterband model for Internet interventions [28]. MORPH Companion will utilize a number of support and social networking features, alongside novel feedback generated using data from 2 commercially available connected health devices (ie, minute-level movement and sitting data from the Fitbit Alta activity monitor, and daily weight data from the Aria scale; each of which will be provided to study participants). The Companion platform will be built as an embedded Web app, appearing to the user as a native app while allowing for compatibility on most mobile devices and rapid updates when necessary. The features of MORPH Companion are detailed below.

#### Core Components of MORPH Companion

**Figure 3:**
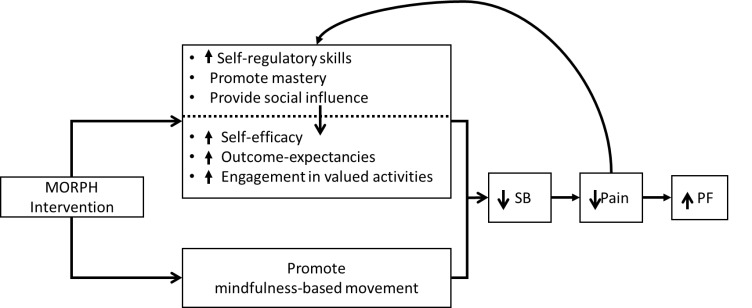
Pathways between intervention support, sedentary behavior, pain, and physical function. MORPH: Mobile Intervention to Reduce Pain and Improve Health; PF: physical function; SB: sedentary behavior.

**Figure 4:**
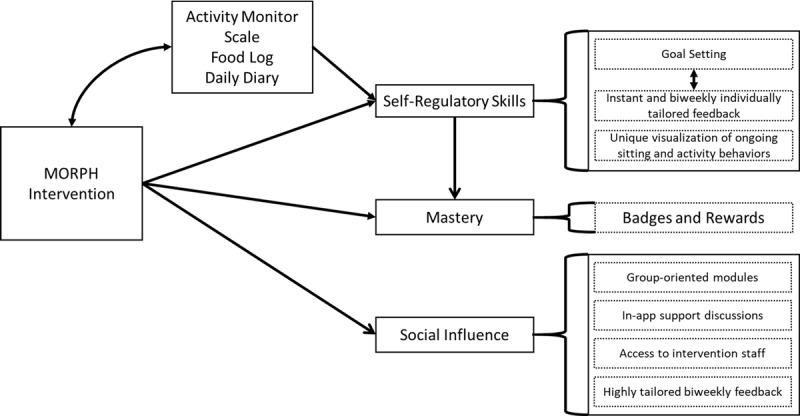
Technology influences on behavioral determinants. MORPH: Mobile Intervention to Reduce Pain and Improve Health.

The goal of MORPH Companion is to provide automated and fully tailored support in a package whose form and function is carefully designed alongside members of the target population, as these are key drivers of app usage within the Ritterband model [28,29]. The MORPH Companion provides immersive intervention content designed to prompt daily movement and to enhance social connections, outcome expectancies, and the participant’s perceived ability to engage in valued activities, each of which are expected to influence an individual’s self-efficacy (ie, one’s situation-specific self-confidence), a primary determinant of behavior change (see Figure 3 [27]). As shown in Figure 4, MORPH Companion specifically targets a number of important social cognitive constructs described in the following sections.

##### Self-Regulatory Skills

**Figure 5:**
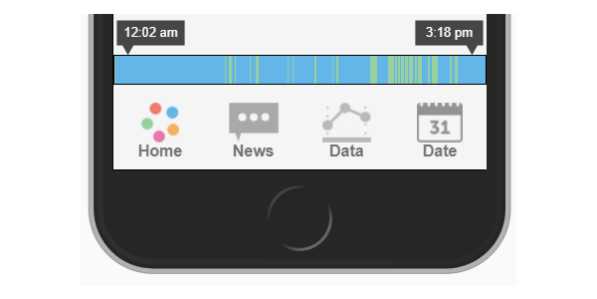
Example of "striated" sitting time feedback. Blue represents sitting; Green represents movement.

MORPH Companion integrates minute-by-minute data streams from commercially available activity monitors, as well as weight data from connected weight scales, to provide real-time visualization of daily body weight, time spent sitting, and postural shift behaviors (eg, see Figure 5). Participants will receive these devices 2 weeks before the start of the intervention, allowing for a 1-week acclimation period [30] and a 1-week baseline data collection period. These data will inform daily and weekly goals and summary feedback, which will be created automatically and then reviewed by research staff on a weekly basis. Importantly, within-day data will be utilized in “striated” sitting time feedback, displaying minute-by-minute sitting and movement behaviors on a daily timeline with the aim of providing insight into the patterns in which sitting and movement are accumulated (Figure 5). We also aim to promote physical activity across the day via *daily movement goals*. *Daily movement goals* (eg, 10,000 steps) are step goals wherein the participant is encouraged to accumulate steps throughout the day by engaging in pleasurable activities of varying intensities. To emphasize day-long movement, the *daily movement goal* is partitioned into 3 periodic movement goals (ie, morning movement, midday movement, evening movement), each allowing the participant to achieve up to 45% of the day’s total goal. In this way, achieving the total movement goal requires the individual to engage in some amount of movement during each of the 3 periods.

In addition to physical activity–oriented goals, postural shift (eg, 70 sit-to-stand transitions across the day), weight (eg, a reduction of 1 pound within the next week, as assessed via the smart scale), and calorie goals (eg, a reduction of approximately 400 kcal/day from weight maintenance requirements) will be crafted or modified weekly by intervention staff and communicated to participants both via email and within the app. Activity monitor data will also be provided to staff to inform these goals. Finally, these data will combine daily diary data, the reward system (each described below), weekly education content, and progress toward weekly goals to automatically generate weekly individualized email feedback, as previous research from our group and others has demonstrated that these regular contacts are important for sustaining app engagement and a sense of social accountability [28,29].

##### Reinforcement of Mastery Experiences and Progress

**Figure 6:**
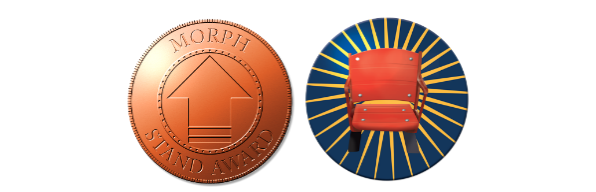
"Bronze Stand Award" (left) and "Stadium Stand" (right) medals.

Setting and achieving intervention goals and receiving both real-time and weekly summary feedback will highlight mastery experiences, which act as the strongest influence on self-efficacy early in the behavior change process [27]. Additionally, our previous research [29] demonstrated that an incentive system of badges and rewards is effective for promoting app engagement and enhancing a number of social cognitive outcomes. These will be integrated throughout the app as a means of complimenting regular feedback to underscore mastery experiences. In addition to the individual goals (eg, daily postural shift goals), participants will also receive monthly group goals (eg, group cumulative step goals). Participants are instructed that achieving these goals release “mastery badges” (eg, “Bronze Stand Award”; see Figure 6), indicating successful movement on the path toward lifestyle behavior change, and they are advised to take a moment to internalize their successes. This is intended to support early and frequent mastery experiences, and in turn promote self-efficacy.

##### Positive Social Influence and Social Connection

Several features of MORPH Companion are designed to promote positive social influence and social connection. These include group challenge modules to foster group collaboration, such as the “Stadium Stand” (see Figure 6), which requires participants to “stand out of the 35,000 seats in the local football stadium” as a group during a 4-week period, and success is accompanied by a unique “team” badge. It is important to note that these group challenges avoid the performance-based climate accompanying competitive “leader-board” systems that inherently highlight individual successes and failures and work against intrinsic motivation and sustained behavior [31]. Rather, they aim to foster positive social bonds and a mastery climate by providing feedback on group successes and the individual’s contribution to that success. Participants will retain access to a social-networking component, wherein weekly discussion topics are provided based upon education content for that week, and participants will be encouraged to share tips and ask for advice. Finally, all participants will be able to indicate discussion content for which interventionist input is desired and will be able to reach intervention staff by phone or email throughout the intervention.

##### Individually Adaptive Content

**Figure 7:**
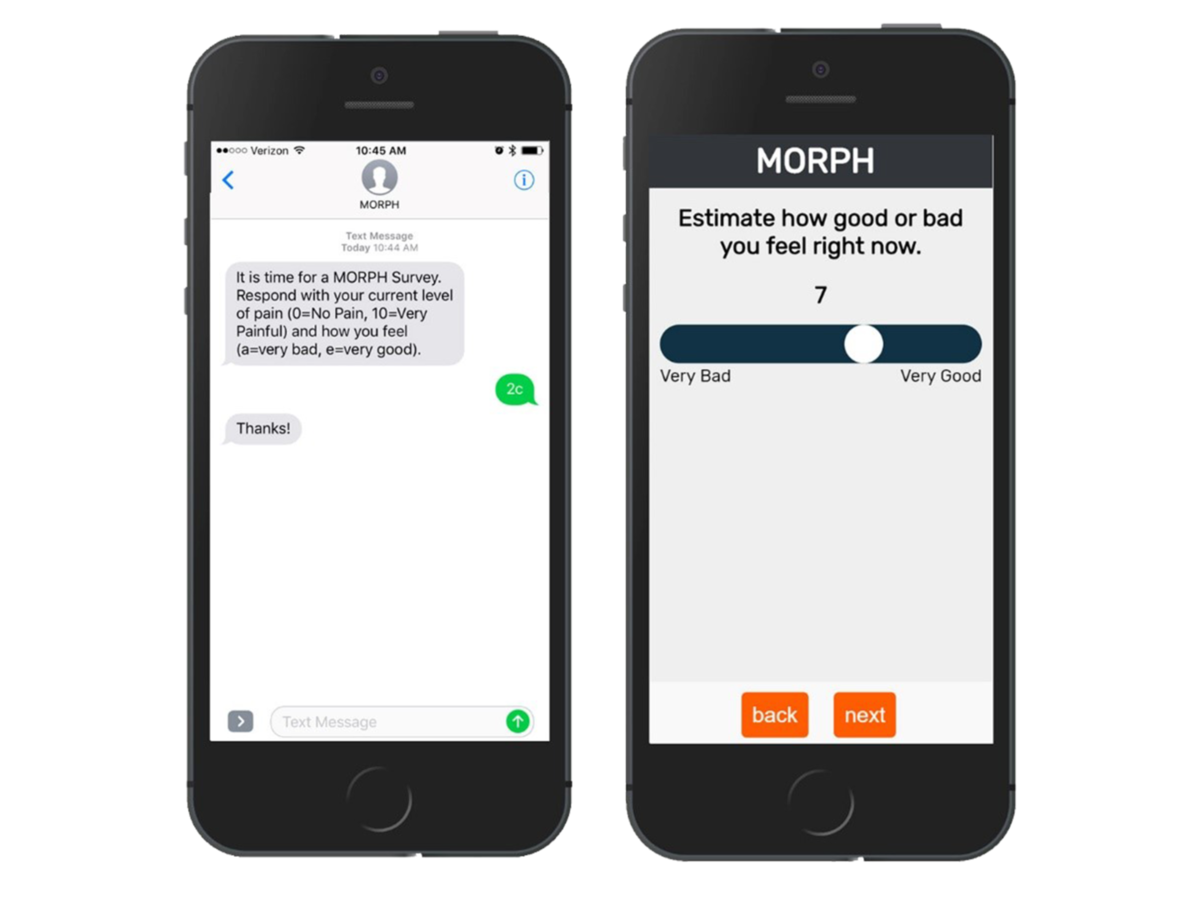
Web-app and text-message ecological momentary assessment options.

Because participants’ pain experiences vary across the day and between days, MORPH Companion also includes adaptive ecological momentary intervention techniques (EMI; the delivery of intervention content in real time [32]). To minimize participant burden often associated with ecological momentary assessment (EMA; the assessment of behaviors and experiences in near real-time and in the context in which they occur [32]), we will use 1-week bursts during weeks 1, 5, and 9. During each burst, participants will receive 6 brief daily assessments delivered via smartphone within 2-hour intervals throughout waking hours. To keep each assessment to a matter of seconds, participants will report only their current level of pain (on an 11-point Likert scale), affect via the Feelings Scale [33], and pain medication use since the last survey. On the first survey of the day, participants will also report the previous night’s sleep duration and quality (assessed via items from the Pittsburgh sleep quality index [34]). Prompts will be delivered via text-message, and during the development phase of MORPH, participants will be presented with 2 response options: (1) via Web app–based surveys using custom EMA software [35] or (2) the option to respond within text messages (see Figure 7). Participants will be tasked with selecting their preferred response method, which will inform the design of the intervention for the pilot randomized controlled trial (RCT; phase II). The primary function of these assessments is to identify daily risk periods: “pain peaks” and “affect troughs.” We expect these 2 variables in particular to contribute to higher levels of SB and unhealthy eating [21,36]. These daily risk periods will be identified in feedback to the participant and will also be used to craft “awareness alerts.” In the initial design of MORPH Companion, these 2 periods will be used to automatically generate 2 daily Fitbit alerts, each triggering a soft vibration to the wrist that will cue the participant to be mindful of their current state and to engage in light to moderate movement and brief mindfulness-based exercises—which are practiced in weekly telecoaching sessions—to improve affect, reduce pain, and prevent overeating or extended sitting. These alarms will be updated automatically with each EMA burst, with staff and participants encouraged to review and revise the timing of daily prompts. Finally, participants will complete 2 daily diary assessments (ie, upon waking, before bed) of daily pain, affect, and medication use when not within an EMA burst period. These will be used to craft feedback and to monitor changes in daily sleep behaviors and ratings of pain and affect in response to the MORPH intervention.

##### Engaging Professionally Developed Session Videos

One of the challenges of presenting material remotely is ensuring that participants engage with and understand important intervention content. To facilitate this process, MORPH will release professionally developed and highly engaging animated cartoons to reinforce and summarize content related to behavioral management of pain, the benefits of day-long movement, and healthy patterns of eating that are discussed in-depth during telecoaching sessions (see [37] for an example video). Participants will receive videos on most weeks of the study and will be encouraged to view the video before the group telecoaching sessions. Participants will also retain access to the videos throughout the duration and following the study.

##### Content of Weight Loss and Sedentary Behavior Intervention

The MORPH Companion app is used within a 9-week telecoaching intervention that is prefaced by 3 weeks of 1.5-hour group sessions for a total intervention spanning 12 weeks. The 3 weeks of initial face-to-face contacts are used to (1) establish the group-mediated nature of the telephone coaching contacts, (2) create connection between group members that will be built upon via the app, (3) teach basic skills needed for successful weight loss and reduced SB, and (4) develop competence in use of the MORPH Companion. The weight loss component targets a 3% to 5% reduction in body weight. The SB component targets (1) increasing the number of postural shifts across the day and (2) increasing the performance of valued activities related to independent living, including balance, strength, and mobility activates that will augment caloric restriction to promote weight loss and increase functional capacities as assessed by the SPPB. The distal goal of the activity component is to increase steps across the day in the range of 5000-10,000 steps based on individual abilities. Individual goals for caloric intake will be prescribed to achieve an energy deficit of approximately 400 kcal/d from daily weight maintenance energy requirements (resting energy expenditure × activity factor of 1.3 for sedentary adults). Participants will maintain either paper or digital food logs (depending on preference), and these will be submitted electronically to study staff on a weekly basis. The lowest caloric goal prescribed will be 1100 kcal/d for women and 1200 kcal/d for men. The structure of the dietary weight loss intervention is based on our extensive experience in community-based weight loss and movement trials for older adults [24,38], whereas the SB intervention is built on our published work with older adults demonstrating that improvements in daily spontaneous physical activity with the aim of reducing sitting improves weight loss and maintenance of weight loss [24]. Sessions will include nutritional education and ongoing assistance in integrating physical activities into daily life, teaching and reinforcement of pain and activity self-regulatory skills, exposure to mindfulness-based stress reduction and pain management, and strategies that optimize social connection. We selected a 12-week intervention for this pilot study for several reasons. First, based on our experience conducting behavioral trials among older adults with osteoarthritis [39], peripheral artery disease [40], and the metabolic syndrome [38,41,42] among others, we believe 12 weeks is sufficient to achieve the weight [24] and physical function [43] goals for this population. Additionally, this duration will allow for the novelty of the daily prompts to diminish, allowing for the examination of the longer-term engagement with the *MORPH* model. These data will be crucial for the design of a future large-scale randomized trial.

#### The MORPH Companion Design Process

Because older adults are likely to have unique needs and desires with regard to app design and functionality, a user-centered design process is essential [44]. Therefore, during the latter 9 months of the initial year of the study period, individual participants (total N=10 or until no substantive changes to the app are requested) will be recruited sequentially to participate in a Think-Aloud protocol. Participants will attend a 1:1 structured session in which they will be instructed to “act as though you are talking to yourself but loud enough for others to hear,” and to attempt to do this as continuously as possible [44,45]. Audio recordings and detailed notes will be made during each session, feedback will be discussed among team members, and modifications made to the app before the next participant completes the protocol. Audio recordings will be transcribed and coded, which will allow for tracking of changes in usability over time. Following this session, in-depth interviews will be conducted to gather information relative to desired features and preferences, and these too will be audio-recorded, transcribed, coded, discussed, and implemented. We have elected to use in-depth interviewing rather than focus groups to acquire detailed reactions to the app and desires for additional app features while reducing participant’s concern for sensitivity of any shared information. Next, the participant will meet with the interventionist to receive one in-person session, including an introduction to the use of MORPH Companion. Following this orientation, participants will be asked to use the app at home for 1 week, piloting all data collection and “adaptive content” components (ie, 1 EMA burst, daily movement prompts, activity monitor wear, and scale setup). After completion of this 1-week study period, participants will return to provide initial reactions to the MORPH Companion, which will be used to refine the interface. Participants will also complete the System Usability Scale (SUS [46]), which collects perceptions of the usability of the app on 10 Likert-type questions (eg, “I found the system cumbersome to use”). The SUS will also be collected from all intervention participants completing the phase-II pilot study.

### Mobile Intervention to Reduce Pain and Improve Health Phase II: The Pilot Study

During the second year of the study period (ie, phase II), we will employ a 2-group randomized controlled design in 30 obese (BMI=30-40 kg/m^2^), low-active older adults with chronic pain, who will be recruited from 2 Wake Forest University Medical system pain clinics and assigned to either a 12-week mHealth+telecoaching intervention (MORPH) or a wait-list control group.

#### Participants

Eligible participants will own an Android or iPhone smartphone, have pain in 2 of 5 areas (ie, back, neck, shoulders, hips, knees) on most days during the previous 3 months [2,47], no contraindication for participation in physical activity and approved for participation by their physician, and will be aged 65-79 years, obese (BMI=30-40 kg/m^2^, weight-stable (ie, no weight loss or gain >5% in the past 6 months), and low-active (ie, engaging in less than 2 days/week of structured physical activity for at least 20 min). Excluded individuals will be unable to walk without assistive devices or will have cognitive impairment as indicated by a Montreal Cognitive Assessment [48] score of less than 22.

**Figure 8:**
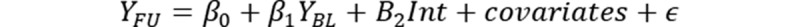
Equation for estimating effect sizes. Y represents the primary outcomes (Short Physical Performance Battery [SPPB] score, Patient-Reported Outcomes Measurement Information System [PROMIS] pain scales); subscripts FU and BL denote measurement at week 12-week follow-up and baseline, respectively; Int represents intervention status; beta represents each regression coefficient; and epsilon represents random error.

#### Primary Outcomes for the Pilot Randomized Controlled Trial

Pain will be assessed using the 3-item PROMIS Pain Intensity Scale and the 8-item Pain Interference Scale [49]. The Pain Intensity Scale tasks participants with rating how much a person hurts on a scale of 1 (had no pain) to 5 (very severe), and the Pain Interference Scale captures the impact of pain on valued areas of an individual’s life (eg, how much did pain interfere with your day-to-day activities) on a scale of 1 (not at all) to 5 (very much). Final scores on both PROMIS measures are given as T-scores on a standardized distribution such that a score of 50 (SD of 10) represents an average score within the United States. Therefore, a score of 60 would represent 1 SD worse pain than average. Physical function will be assessed using the SPPB [50]. This test of lower-extremity function consists of 4-min walk at usual pace, a timed repeated chair stand, and 3 increasingly difficult standing balance tests. Each measure is assigned a categorical score ranging from 0 (inability to complete the test) to 4 (best performance) resulting in a final score of 0 to 12.

#### Secondary Outcomes for the Randomized Controlled Trial

Body mass will be assessed using the same scale, which is accurate to ±100 g and calibrated weekly. In addition to activity data collected via the Fitbit device, sedentary and light activity minutes and transitions will be assessed using ActivPal activity monitors (PAL Technologies Ltd, Glasgow, UK) worn at the thigh for 7 consecutive days before the start of the study and again during week 12. We will assess the number of transitions from SB to non-SB and number of daily steps. Feasibility will be determined based upon (1) whether change in primary outcomes was observed; (2) the extent to which participants were retained in the study; and (3) the extent to which participants utilized the study application. Specifically, the participants will be asked to try to use the app daily to ensure Fitbit data have automatically synchronized and to view progress toward daily transition goals, and we will examine within-person trends in weekly app use across 12 weeks.

#### Analysis Plan

First, simple descriptive statistics will be utilized to evaluate usability (eg, SUS scores) and feasibility of the intervention (eg, application accesses per week throughout the study period). With regard to the phase-II pilot RCT, the analytic goals are to (1) estimate the effect size of the intervention such that the results can be used to power a future larger RCT and (2) explore appropriate methods and models for analyzing the EMA/EMI data. We plan to estimate the effect size using the generalized linear model depicted in Figure 8. Due to the small sample size, we may include important covariates (eg, gender, age) if the two arms happen to be highly imbalanced. In case covariates are used, effect sizes will be estimated using the adjusted values from the above equation. We also plan to conduct similar analyses of secondary outcomes of the RCT (eg, weight loss and SB) using the aforementioned model, applying the appropriate model link function if the secondary outcome is not continuous. We also recognize the potential value of the intensively collected EMA data. As such, we will conduct exploratory analyses to determine if preliminary evidence supports the cross-lagged reciprocal relationship between pain and self-efficacy (Figure 2). The exploratory analysis is expected to inform the analytic plan for a future larger study.

### Potential Limitations

There are 2 key limitations we will attempt to address during the design and development phase of MORPH and MORPH Companion.

#### Participant Burden

When possible, connected sensors are used (ie, activity monitor, scale) to passively collect data, and we are collecting EMAs in bursts to minimize assessment burden. Importantly, as we have found that tying the collection of these data to valued participant outcomes reduced perceived burden, EMA and sensor data are used to provide tailored feedback to the participant and in-app rewards and badges.

#### Discomfort With Technology

Although many older adults use mobile technologies, many do not. Moreover, those who do use the technology often use fewer features relative to younger populations. In this regard, we have had success with a brief but immersive orientation session that occurs when activity monitors are distributed. Participants receive a “dummy” app with all features enabled. They work with the interventionist to navigate the app’s features. They receive text message prompts and complete several EMAs. This process is continued until the participant is content with their ability to access and navigate the app interface. Importantly, we aim to minimize these 2 limitations during phase I by receiving and iterating on participant feedback related to the app.

## Results

Phase 1 recruitment is ongoing as of December 2017.

## Discussion

Chronic pain often acts as a significant threat to an older adult’s physical functioning and independent living, and traditional treatment options carry myriad risks. MORPH Companion development is underway, with recruitment beginning in March of 2018. The MORPH package brings together a strong body of evidence using group-based behavioral intervention designs with contemporary mHealth principles, allowing for intervention when and where it matters the most. Given the ubiquity of smartphone devices and the popularity of consumer activity and weight monitors, the results of this study may serve to inform the development of scalable, socially driven movement interventions.

## Abbreviations

BMI: body mass index
EMA: ecological momentary assessment
EMI: ecological momentary intervention
MORPH: Mobile Intervention to Reduce Pain and Improve Health
MVPA: moderate to vigorous physical activity
PROMIS: Patient-Reported Outcomes Measurement Information System
RCT: randomized controlled trial
SB: sedentary behavior
SPPB: Short Physical Performance Battery
SUS: System Usability Scale
